# Comparison crystal structure conformations of two structurally related biphenyl analogues: 4,4′-bis­[3-(pyrrolidin-1-yl)prop-1-yn-1-yl]-1,1′-biphenyl and 4,4′-bis­{3-[(*S*)-2-methyl­pyrrolidin-1-yl]prop-1-yn-1-yl}-1,1′-biphen­yl

**DOI:** 10.1107/S2056989015016163

**Published:** 2015-09-12

**Authors:** Anqi Wan, Narsimha Reddy Penthala, E. Kim Fifer, Sean Parkin, Peter A. Crooks

**Affiliations:** a Dept. of Pharm. Sciences, College of Pharmacy, University of Arkansas for Medical Sciences, Little Rock, AR 72205, USA; b Dept. of Chemistry, University of Kentucky, Lexington KY 40506, USA

**Keywords:** crystal structure, bis-tertiary ammonium salt, biphenyl ring, pyrolidine ring

## Abstract

The title compounds, C_26_H_28_N_2_, (I), and C_28_H_32_N_2_, (II), were designed based on the structure of the potent α9α10 nicotinic acetyl­choline receptor antagonist ZZ161C {1,1′-[[1,1′-biphen­yl]-4,4′-diylbis(prop-2-yne-3,1-di­yl)]bis­(3,4-di­methyl­pyridin-1-ium) bromide}. In order to improve the druglikeness properties of ZZ161C for potential oral administration, the title compounds (I) and (II) were prepared by coupling 4,4′-bis­(3-bromo­prop-1-yn-1-yl)-1,1′-biphenyl with pyrrol­idine, (I), and (*S*)-2-methyl­pyrrolidine, (II), respectively, in aceto­nitrile at room temperature. The asymmetric unit of (I) contains two half mol­ecules that each sit on sites of crystallographic inversion. As a result, the biphenyl ring systems in compound (I) are coplanar. The biphenyl ring system in compound (II), however, has a dihedral angle of 28.76 (11)°. In (I), the two independent mol­ecules differ in the orientation of the pyrrolidine ring (the nitro­gen lone pair points towards the biphenyl rings in one mol­ecule, but away from the rings in the other). The torsion angles about the ethynyl groups between the planes of the phenyl rings and the pyrrolidine ring *N* atoms are 84.15 (10) and −152.89 (10)°. In compound (II), the corresponding torsion angles are 122.0 (3) and 167.0 (3)°, with the nitro­gen lone pairs at both ends of the mol­ecule directed away from the central biphenyl rings.

## Abstract 2

The crystal structures of the two title compounds each display the chair conformation of their piperidine rings. In 4,4′-bis­[3-(pyrrolidin-1-yl)prop-1-yn-1-yl]-1,1′-biphenyl, the biphenyl rings are coplanar because the mol­ecules sit on crystallographic centres of inversion. In 4,4′-bis­{3-[(*S*)-2-methyl­pyrrolidin-1-yl]prop-1-yn-1-yl}-1,1′-biphenyl, the biphenyl ring system has a twisted conformation with a dihedral angle of 28.76 (11)°.

## Chemical context   

The title compounds (I) and (II) are structural analogue precursors of the bis-quaternary ammonium salt, ZZ161C {1′-[(1,1′-biphen­yl)-4,4′-diylbis(prop-2-yne-3,1-di­yl)]bis­(3,4-di­methyl­pyridin-1-ium) bromide}, designed to improve druglikeness properties. ZZ161C is a potent and selective nicotinic acetyl­choline receptor antagonist for α9α10 subunits (Zheng *et al.*, 2007 ▸), and has shown analgesic effects in various animal pain models (Wala *et al.*, 2012 ▸). The terminal aza-aromatic rings were replaced by pyrrolidine and (*S*)-2-methyl­pyrrolidine moieties in compounds (I) and (II), respectively. We report here the single-crystal X-ray structures of (I) and (II) to determine the conformations of these compounds.

## Structural commentary   

**Figure 1:**
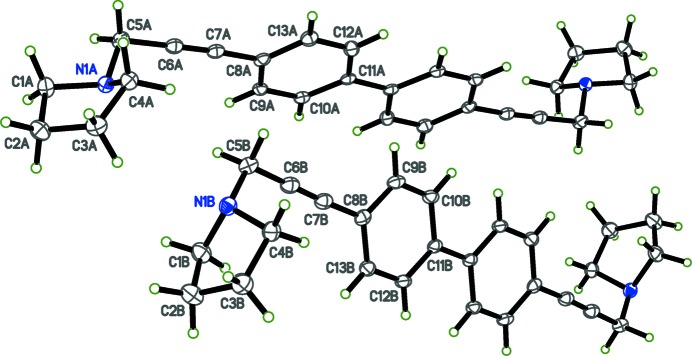
The mol­ecular structure of (I), with ellipsoids drawn at the 50% probability level.

The title compounds, (I) and (II) are shown in Figs. 1 ▸ and 2 ▸, respectively. X-ray crystallographic studies were carried out in order to determine the geometry of the biphenyl ring systems, as well as to obtain more detailed information about the conformation of the pyrrolidino headgroups. Structure (I) is triclinic, space group *P* 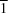, while crystal (II) is monoclinic, space group *P*2_1_.
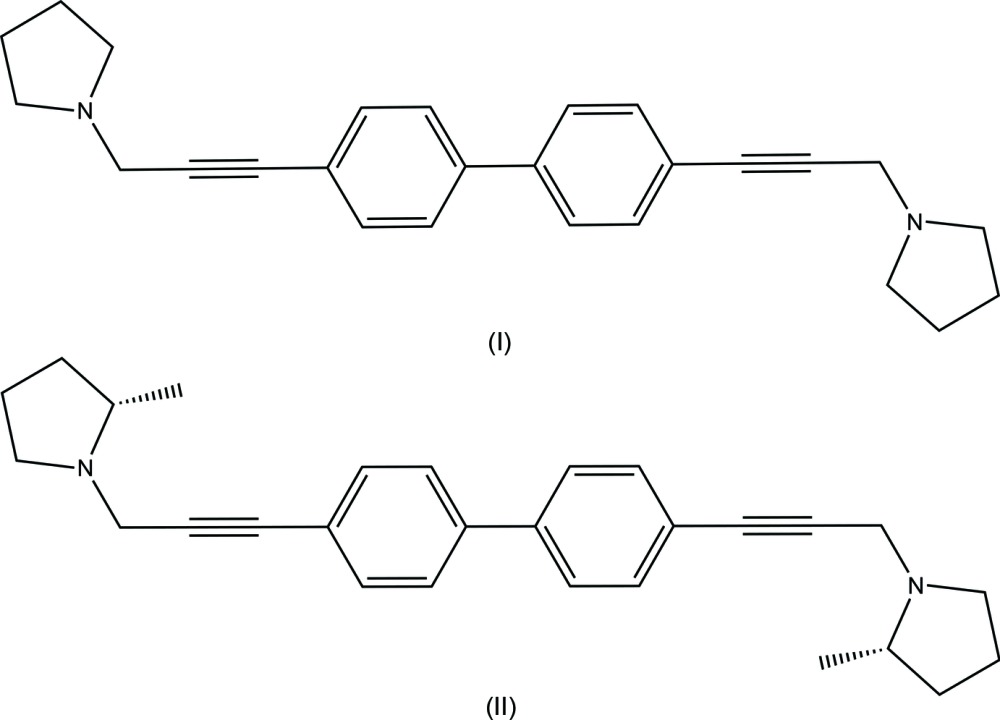


 In each compound, individual bond lengths and angles are unremarkable. For compound (I), the asymmetric unit contains two half mol­ecules (denoted A and B in Fig. 1 ▸) such that the biphenyl rings straddle crystallographic inversion centres. As a result, the biphenyl groups are coplanar. In compound (II), however, the biphenyl rings (C9–C14) and (C15–C20) are non-coplanar, with a dihedral angle of 28.76 (11)°. In crystals of (I), the two independent mol­ecules differ in the orientation of the pyrrolidine ring. In mol­ecule *A*, the nitro­gen lone pair points inward towards the biphenyl rings, but in mol­ecule *B* the nitro­gen lone pair is directed away from the rings). The torsion angles about the ethynyl groups between the planes of the phenyl rings and the pyrrolidine ring *N* atoms are 84.15 (10)° and −152.89 (10)° (defined by atoms N1*A*—C5*A*—C8*A*—C9*A* and N1*B*—C5*B*—C8*B*—C9*B*, respectively). In compound (II), the corresponding torsion angles are 122.0 (3)° and 167.0 (3)° (defined by atoms N1—C6—C9—C14 and N2—C23—C18—C17, respectively), with the nitro­gen lone pair directed away from the biphenyl rings at both ends of the mol­ecule.

## Supra­molecular features   

Aside from weak van der Waals inter­actions, there are no noteworthy inter­molecular contacts in either (I) or (II).

## Database survey   

A search of the November 2014 release of the Cambridge Structure Database (Groom & Allen, 2014 ▸), with updates through May 2015, using the program *Mogul* (Bruno *et al.*, 2004 ▸) for 4,4′ substituted biphenyl fragments was conducted. The search was restricted to non-organometallic, solvent-free structures with *R* < 5% and Cl as the heaviest element. There were over 1000 matches, which gave a bimodal distribution of biphenyl torsion angles with a tight peak at 0° and a broader peak centred at 30°. The biphenyl torsion angles in (I) and (II) are thus not unusual.

## Synthesis and crystallization   

**Synthetic procedures:** Compound (I), 3,3′-([1,1′-biphen­yl]-4,4′-di­yl)bis (prop-2-yn-1-ol) was synthesized by coupling 1,2,4,5-tetra­iodo­benzene with 4-pentyn-1-ol in the presence of bis-(tri­phenyl­phosphine)palladium(II)dichloride and copper(I) iodide as catalysts. A mixture of 1,2,4,5-tetra­iodo­benzene, 4-pentyn-1-ol, bis-(tri­phenyl­phosphine)palla­dium(II)dichloride and copper(I) iodide was stirred at room temperature for 24 h under argon. The obtained 3,3′-([1,1′-biphen­yl]-4,4′-di­yl)bis­(prop-2-yn-1-ol) was converted to 4,4′-bis-(3-bromo­prop-1-yn-1-yl)-1,1′-biphenyl using bromo­methane and tri­phenyl­phosphine in anhydrous methyl­ene chloride at room temperature. To a suspension of 4,4′-bis­(3-bromo­prop-1-yn-1-yl)-1,1′-biphenyl (100.0 mg, 0.26 mmol) in aceto­nitrile (7 mL) was added pyrrolidine (55.4 mg, 0.78 mmol) and the reaction mixture was stirred for 2 h at room temperature to obtain compound (I). Aceto­nitrile was removed from the reaction mixture under reduced pressure and the resulting residue was partitioned between water and di­chloro­methane. The organic layers were collected, combined, dried over anhydrous sodium sulfate, filtered, and the filtrate concentrated under reduced pressure. The resulting crude sample of (I) was purified by column chromatography (di­chloro­methane/methanol, 100:3) (yield: 80%). Compound (II) was prepared using the same experimental conditions as (I) but utilizing (*S*)-2-methyl­pyrrolidine (66.3 mg, 0.78 mmol) instead of pyrrolidine. Column chromatography (di­chloro­methane/methanol 100:3) was then used for purification of (II) (yield: 80%).

**Crystallization:** Yellow crystals of compounds (I) and (II) suitable for X-ray analysis were grown from a mixture of di­chloro­methane/methanol (2:1) by slow evaporation of the solution at room temperature over 24 h.

**Compound (I)**

^1^H NMR (400 Mz, CDCl_3_): δ 7.49 (*q*, 8H), 3.67 (*s*, 4H), 2.75 (*s*, 8H), 1.86 (*s*, 8H) p.p.m.

^13^C NMR (100 Mz, CDCl_3_): δ 139.94. 132.19, 126.77, 122.32, 85.67, 84.55, 52.65, 43.85, 23.83 p.p.m.

**Compound (II)**

^1^H NMR (400 Mz, CDCl_3_): δ 7.21 (*q*, 8H), 3.69 (*dd*, 4H), 3.16–3.11 (*m*, 2H), 2.69–2.59 (*m*, 4H), 2.01–1.43 (*m*, 8H), 1.15 (*d*, 6H) p.p.m.

^13^C NMR (100 Mz, CDCl_3_): δ 139.86, 132.18, 126.74, 122.43, 85.53, 84.61, 57.31, 53.00, 41.18, 32.79, 21.55, 18.51 p.p.m.

## Refinement details   

Crystal data, data collection and structure refinement details are summarized in Table 1 ▸. In both structures, H atoms were found in difference Fourier maps, but subsequently included in the refinement using riding models. Constrained distances were set to 0.95 Å (C_sp2_H), 0.98 Å [*R*CH_3_, (II) only], 0.99 Å (*R*_2_CH_2_) and 1.00 Å (*R*_3_CH). *U*_iso_(H) parameters were set to values of either 1.2*U*_eq_ or 1.5*U*_eq_ [*R*CH_3_ in (II) only] of the attached atom.

In (II), the Flack parameter, *x* = −0.3 (10) is indeterminate, which is to be expected for a light-atom structure refined against Mo *Kα* data. However, the synthesis used pure (*S*)-2-methyl­pyrrolidine, so the absolute configuration for the model of (II) was dictated by the synthesis.

Refinement progress was checked using *PL*ATON (Spek, 2009 ▸) and by an *R*-tensor (Parkin, 2000 ▸). The final models were further checked with the IUCr utility *checkCIF*.

## Supplementary Material

Crystal structure: contains datablock(s) global, I, II. DOI: 10.1107/S2056989015016163/hg5457sup1.cif

Structure factors: contains datablock(s) I. DOI: 10.1107/S2056989015016163/hg5457Isup2.hkl

Structure factors: contains datablock(s) II. DOI: 10.1107/S2056989015016163/hg5457IIsup3.hkl

Supporting information file. DOI: 10.1107/S2056989015016163/hg5457Isup4.cml

Supporting information file. DOI: 10.1107/S2056989015016163/hg5457IIsup5.cml

CCDC references: 1421219, 1421218

Additional supporting information:  crystallographic information; 3D view; checkCIF report

## Figures and Tables

**Figure 2:**
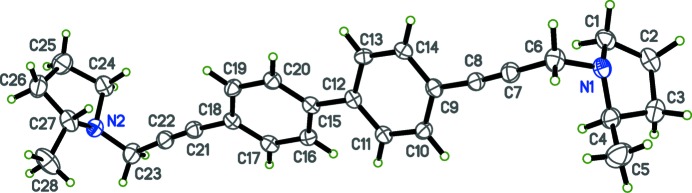
The mol­ecular structure of (II), with ellipsoids drawn at the 50% probability level.

**Table 1:** Experimental details

	(I)	(II)
Crystal data		
Chemical formula	C_26_H_28_N_2_	C_28_H_32_N_2_
*M*_r_	368.50	396.55
Crystal system, space group	Triclinic, *P* 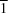	Monoclinic, *P*2_1_
Temperature (K)	90	90
*a*, *b*, *c* (Å)	6.2100 (1), 10.3089 (2), 16.3082 (3)	8.1411 (4), 7.3080 (4), 18.9840 (9)
α, β, γ (°)	86.317 (1), 81.202 (1), 76.671 (1)	90, 98.177 (3), 90
*V* (Å^3^)	1003.49 (3)	1117.97 (10)
*Z*	2	2
Radiation type	Cu *K*α	Mo *K*α
μ (mm^−1^)	0.54	0.07
Crystal size (mm)	0.23 × 0.19 × 0.10	0.41 × 0.35 × 0.08

Data collection		
Diffractometer	Bruker X8 Proteum	Nonius KappaCCD
Absorption correction	Multi-scan (*SADABS*; Krause *et al.*, 2015 ▸)	Multi-scan (*SADABS*; Krause *et al.*, 2015 ▸)
*T*_min_, *T*_max_	0.811, 0.929	0.791, 0.971
No. of measured, independent and observed [*I* > 2σ(*I*)] reflections	13692, 3586, 3451	15874, 4705, 3548
*R*_int_	0.044	0.085
(sin θ/λ)_max_ (Å^−1^)	0.602	0.650

Refinement		
*R*[*F* ^2^ > 2σ(*F* ^2^)], *wR*(*F* ^2^), *S*	0.039, 0.107, 1.03	0.054, 0.144, 1.05
No. of reflections	3586	4705
No. of parameters	254	273
No. of restraints	0	1
H-atom treatment	H-atom parameters constrained	H-atom parameters constrained
Δρ_max_, Δρ_min_ (e Å^−3^)	0.22, −0.20	0.30, −0.19
Absolute structure	–	Flack *x* parameter was determined using 1205 quotients of the form [(*I* ^+^)−(*I* ^−^)]/[(*I* ^+^)+(*I* ^−^)] (Parsons *et al.*, 2013 ▸)
Absolute structure parameter	–	−0.3 (10)

Computer programs: *APEX2* and *SAINT* (Bruker, 2006 ▸), *COLLECT* (Nonius, 1998 ▸), *SCALEPACK* and *DENZO-SMN* (Otwinowski & Minor, 2006 ▸), *SHELXS97*, *XP in *SHELXTL** and *SHELX* (Sheldrick, 2008 ▸) (Sheldrick, 2008 ▸), *SHELXL2014/6* and *SHELXL2014* (Sheldrick, 2015 ▸) and *CIFFIX* (Parkin, 2013 ▸).

## supplementary crystallographic information

### (I) 4,4'-Bis[3-(pyrrolidin-1-yl)prop-1-yn-1-yl]-1,1'-biphenyl Crystal data

**Table:**

C_26_H_28_N_2_	*Z* = 2
*M**_r_* = 368.50	*F*(000) = 396
Triclinic, *P*1	*D*_x_ = 1.220 Mg m^−^^3^
*a* = 6.2100 (1) Å	Cu *K*α radiation, λ = 1.54178 Å
*b* = 10.3089 (2) Å	Cell parameters from 9977 reflections
*c* = 16.3082 (3) Å	θ = 2.7–68.2°
α = 86.317 (1)°	µ = 0.54 mm^−^^1^
β = 81.202 (1)°	*T* = 90 K
γ = 76.671 (1)°	Shard, colourless
*V* = 1003.49 (3) Å^3^	0.23 × 0.19 × 0.10 mm

### (I) 4,4'-Bis[3-(pyrrolidin-1-yl)prop-1-yn-1-yl]-1,1'-biphenyl Data collection

**Table:**

Bruker X8 Proteum diffractometer	3586 independent reflections
Radiation source: fine-focus rotating anode	3451 reflections with *I* > 2σ(*I*)
Detector resolution: 5.6 pixels mm^-1^	*R*_int_ = 0.044
φ and ω scans	θ_max_ = 68.2°, θ_min_ = 2.7°
Absorption correction: multi-scan (*SADABS*; Krause *et al.*, 2015)	*h* = −7→7
*T*_min_ = 0.811, *T*_max_ = 0.929	*k* = −12→6
13692 measured reflections	*l* = −19→17

### (I) 4,4'-Bis[3-(pyrrolidin-1-yl)prop-1-yn-1-yl]-1,1'-biphenyl Refinement

**Table:**

Refinement on *F*^2^	Secondary atom site location: difference Fourier map
Least-squares matrix: full	Hydrogen site location: difference Fourier map
*R*[*F*^2^ > 2σ(*F*^2^)] = 0.039	H-atom parameters constrained
*wR*(*F*^2^) = 0.107	*w* = 1/[σ^2^(*F*_o_^2^) + (0.0577*P*)^2^ + 0.3125*P*] where *P* = (*F*_o_^2^ + 2*F*_c_^2^)/3
*S* = 1.03	(Δ/σ)_max_ < 0.001
3586 reflections	Δρ_max_ = 0.22 e Å^−^^3^
254 parameters	Δρ_min_ = −0.20 e Å^−^^3^
0 restraints	Extinction correction: *SHELXL2014/6* (Sheldrick, 2015), Fc^*^=kFc[1+0.001xFc^2^λ^3^/sin(2θ)]^-1/4^
Primary atom site location: structure-invariant direct methods	Extinction coefficient: 0.0061 (11)

### (I) 4,4'-Bis[3-(pyrrolidin-1-yl)prop-1-yn-1-yl]-1,1'-biphenyl Special details

**Table:**

Geometry. All e.s.d.'s (except the e.s.d. in the dihedral angle between two l.s. planes) are estimated using the full covariance matrix. The cell e.s.d.'s are taken into account individually in the estimation of e.s.d.'s in distances, angles and torsion angles; correlations between e.s.d.'s in cell parameters are only used when they are defined by crystal symmetry. An approximate (isotropic) treatment of cell e.s.d.'s is used for estimating e.s.d.'s involving l.s. planes.
Refinement. Refinement progress was checked using *PLATON* (Spek, 2009) and by an *R*-tensor (Parkin, 2000). The final model was further checked with the IUCr utility *checkCIF*.

### (I) 4,4'-Bis[3-(pyrrolidin-1-yl)prop-1-yn-1-yl]-1,1'-biphenyl Fractional atomic coordinates and isotropic or equivalent isotropic displacement parameters (Å^2^)

**Table:**

	*x*	*y*	*z*	*U*_iso_*/*U*_eq_	
N1A	0.90671 (15)	0.09727 (9)	0.80861 (5)	0.0163 (2)	
C1A	1.11598 (18)	0.01531 (11)	0.83141 (7)	0.0210 (3)	
H1A1	1.2346	0.0010	0.7828	0.025*	
H1A2	1.0951	−0.0723	0.8555	0.025*	
C2A	1.1724 (2)	0.09853 (12)	0.89584 (7)	0.0238 (3)	
H2A1	1.2687	0.1582	0.8692	0.029*	
H2A2	1.2500	0.0406	0.9381	0.029*	
C3A	0.9430 (2)	0.17995 (12)	0.93527 (7)	0.0226 (3)	
H3A1	0.9140	0.1575	0.9953	0.027*	
H3A2	0.9359	0.2769	0.9280	0.027*	
C4A	0.77498 (18)	0.13928 (11)	0.88842 (7)	0.0187 (2)	
H4A1	0.7159	0.0651	0.9180	0.022*	
H4A2	0.6486	0.2155	0.8810	0.022*	
C5A	0.79816 (19)	0.02600 (11)	0.75852 (7)	0.0190 (2)	
H5A1	0.7276	−0.0379	0.7948	0.023*	
H5A2	0.9128	−0.0257	0.7167	0.023*	
C6A	0.62761 (18)	0.11598 (10)	0.71603 (7)	0.0175 (2)	
C7A	0.49421 (18)	0.18772 (10)	0.67760 (6)	0.0168 (2)	
C8A	0.34803 (18)	0.27515 (10)	0.62731 (7)	0.0160 (2)	
C9A	0.43797 (18)	0.32491 (10)	0.55121 (7)	0.0160 (2)	
H9A	0.5942	0.2993	0.5331	0.019*	
C10A	0.30252 (18)	0.41090 (10)	0.50192 (6)	0.0156 (2)	
H10A	0.3677	0.4427	0.4503	0.019*	
C11A	0.07141 (17)	0.45231 (9)	0.52628 (6)	0.0145 (2)	
C12A	−0.01727 (18)	0.39888 (10)	0.60194 (7)	0.0177 (2)	
H12A	−0.1738	0.4232	0.6197	0.021*	
C13A	0.11676 (19)	0.31184 (11)	0.65140 (7)	0.0182 (2)	
H13A	0.0513	0.2769	0.7020	0.022*	
N1B	0.65099 (16)	0.52088 (9)	0.86778 (6)	0.0188 (2)	
C1B	0.84890 (19)	0.56666 (12)	0.82796 (7)	0.0235 (3)	
H1B1	0.8123	0.6326	0.7824	0.028*	
H1B2	0.9688	0.4911	0.8056	0.028*	
C2B	0.9172 (2)	0.63028 (13)	0.89876 (8)	0.0271 (3)	
H2B1	0.9916	0.7035	0.8777	0.033*	
H2B2	1.0199	0.5635	0.9293	0.033*	
C3B	0.6947 (2)	0.68421 (12)	0.95488 (7)	0.0253 (3)	
H3B1	0.7020	0.6498	1.0127	0.030*	
H3B2	0.6594	0.7829	0.9546	0.030*	
C4B	0.51897 (19)	0.63353 (11)	0.91723 (7)	0.0205 (3)	
H4B1	0.4102	0.6041	0.9612	0.025*	
H4B2	0.4373	0.7036	0.8818	0.025*	
C5B	0.52980 (19)	0.47407 (11)	0.80985 (7)	0.0204 (3)	
H5B1	0.4064	0.4386	0.8422	0.024*	
H5B2	0.6326	0.3993	0.7790	0.024*	
C6B	0.43522 (19)	0.57591 (11)	0.74928 (7)	0.0199 (3)	
C7B	0.35385 (19)	0.66217 (11)	0.70308 (7)	0.0190 (3)	
C8B	0.25131 (18)	0.76103 (10)	0.64637 (6)	0.0170 (2)	
C9B	0.06171 (19)	0.74642 (11)	0.61458 (7)	0.0180 (2)	
H9B	−0.0018	0.6720	0.6322	0.022*	
C10B	−0.03435 (18)	0.83874 (10)	0.55795 (7)	0.0173 (2)	
H10B	−0.1620	0.8259	0.5369	0.021*	
C11B	0.05162 (17)	0.95096 (10)	0.53077 (6)	0.0155 (2)	
C12B	0.23930 (18)	0.96591 (10)	0.56433 (7)	0.0175 (2)	
H12B	0.3002	1.0417	0.5481	0.021*	
C13B	0.33793 (18)	0.87324 (11)	0.62041 (7)	0.0180 (2)	
H13B	0.4658	0.8858	0.6415	0.022*	

### (I) 4,4'-Bis[3-(pyrrolidin-1-yl)prop-1-yn-1-yl]-1,1'-biphenyl Atomic displacement parameters (Å^2^)

**Table:**

	*U*^11^	*U*^22^	*U*^33^	*U*^12^	*U*^13^	*U*^23^
N1A	0.0170 (5)	0.0163 (4)	0.0157 (5)	−0.0032 (4)	−0.0031 (4)	−0.0010 (3)
C1A	0.0179 (5)	0.0229 (6)	0.0211 (6)	−0.0011 (4)	−0.0042 (4)	−0.0021 (4)
C2A	0.0220 (6)	0.0290 (6)	0.0221 (6)	−0.0076 (5)	−0.0056 (5)	−0.0016 (5)
C3A	0.0264 (6)	0.0233 (6)	0.0192 (6)	−0.0064 (5)	−0.0039 (5)	−0.0032 (4)
C4A	0.0191 (5)	0.0188 (5)	0.0173 (5)	−0.0037 (4)	−0.0004 (4)	−0.0007 (4)
C5A	0.0225 (6)	0.0151 (5)	0.0200 (5)	−0.0034 (4)	−0.0059 (4)	−0.0013 (4)
C6A	0.0200 (5)	0.0162 (5)	0.0174 (5)	−0.0064 (4)	−0.0019 (4)	−0.0028 (4)
C7A	0.0207 (5)	0.0143 (5)	0.0169 (5)	−0.0067 (4)	−0.0021 (4)	−0.0028 (4)
C8A	0.0209 (6)	0.0111 (5)	0.0175 (5)	−0.0051 (4)	−0.0047 (4)	−0.0032 (4)
C9A	0.0162 (5)	0.0133 (5)	0.0198 (5)	−0.0053 (4)	−0.0021 (4)	−0.0034 (4)
C10A	0.0190 (5)	0.0124 (5)	0.0163 (5)	−0.0062 (4)	−0.0012 (4)	−0.0013 (4)
C11A	0.0186 (5)	0.0099 (5)	0.0165 (5)	−0.0055 (4)	−0.0025 (4)	−0.0037 (4)
C12A	0.0170 (5)	0.0168 (5)	0.0186 (5)	−0.0035 (4)	0.0002 (4)	−0.0017 (4)
C13A	0.0216 (6)	0.0167 (5)	0.0162 (5)	−0.0055 (4)	−0.0008 (4)	−0.0002 (4)
N1B	0.0192 (5)	0.0182 (5)	0.0180 (5)	−0.0030 (4)	−0.0013 (4)	−0.0010 (4)
C1B	0.0199 (6)	0.0284 (6)	0.0214 (6)	−0.0060 (5)	0.0013 (4)	−0.0028 (5)
C2B	0.0230 (6)	0.0339 (7)	0.0262 (6)	−0.0102 (5)	−0.0022 (5)	−0.0038 (5)
C3B	0.0263 (6)	0.0267 (6)	0.0233 (6)	−0.0063 (5)	−0.0026 (5)	−0.0054 (5)
C4B	0.0201 (6)	0.0212 (6)	0.0190 (5)	−0.0029 (4)	−0.0006 (4)	−0.0028 (4)
C5B	0.0242 (6)	0.0171 (5)	0.0204 (6)	−0.0059 (4)	−0.0027 (4)	−0.0002 (4)
C6B	0.0224 (6)	0.0192 (6)	0.0190 (6)	−0.0073 (4)	−0.0013 (4)	−0.0030 (4)
C7B	0.0214 (6)	0.0180 (5)	0.0181 (5)	−0.0062 (4)	−0.0001 (4)	−0.0041 (4)
C8B	0.0201 (5)	0.0153 (5)	0.0146 (5)	−0.0026 (4)	0.0008 (4)	−0.0046 (4)
C9B	0.0230 (6)	0.0141 (5)	0.0181 (5)	−0.0075 (4)	0.0004 (4)	−0.0037 (4)
C10B	0.0186 (5)	0.0157 (5)	0.0190 (5)	−0.0061 (4)	−0.0014 (4)	−0.0047 (4)
C11B	0.0171 (5)	0.0131 (5)	0.0159 (5)	−0.0037 (4)	0.0016 (4)	−0.0054 (4)
C12B	0.0184 (5)	0.0144 (5)	0.0204 (5)	−0.0067 (4)	0.0003 (4)	−0.0032 (4)
C13B	0.0173 (5)	0.0179 (5)	0.0191 (5)	−0.0044 (4)	−0.0012 (4)	−0.0047 (4)

### (I) 4,4'-Bis[3-(pyrrolidin-1-yl)prop-1-yn-1-yl]-1,1'-biphenyl Geometric parameters (Å, º)

**Table:**

N1A—C4A	1.4609 (13)	N1B—C5B	1.4613 (14)
N1A—C5A	1.4612 (13)	N1B—C1B	1.4625 (15)
N1A—C1A	1.4637 (14)	N1B—C4B	1.4663 (14)
C1A—C2A	1.5264 (15)	C1B—C2B	1.5228 (16)
C1A—H1A1	0.9900	C1B—H1B1	0.9900
C1A—H1A2	0.9900	C1B—H1B2	0.9900
C2A—C3A	1.5442 (16)	C2B—C3B	1.5430 (17)
C2A—H2A1	0.9900	C2B—H2B1	0.9900
C2A—H2A2	0.9900	C2B—H2B2	0.9900
C3A—C4A	1.5283 (15)	C3B—C4B	1.5329 (16)
C3A—H3A1	0.9900	C3B—H3B1	0.9900
C3A—H3A2	0.9900	C3B—H3B2	0.9900
C4A—H4A1	0.9900	C4B—H4B1	0.9900
C4A—H4A2	0.9900	C4B—H4B2	0.9900
C5A—C6A	1.4667 (15)	C5B—C6B	1.4775 (15)
C5A—H5A1	0.9900	C5B—H5B1	0.9900
C5A—H5A2	0.9900	C5B—H5B2	0.9900
C6A—C7A	1.2012 (16)	C6B—C7B	1.1987 (16)
C7A—C8A	1.4369 (15)	C7B—C8B	1.4350 (15)
C8A—C9A	1.3976 (15)	C8B—C9B	1.3986 (16)
C8A—C13A	1.3986 (16)	C8B—C13B	1.4003 (16)
C9A—C10A	1.3825 (15)	C9B—C10B	1.3815 (15)
C9A—H9A	0.9500	C9B—H9B	0.9500
C10A—C11A	1.4017 (15)	C10B—C11B	1.4023 (15)
C10A—H10A	0.9500	C10B—H10B	0.9500
C11A—C12A	1.4049 (15)	C11B—C12B	1.4037 (15)
C11A—C11A^i^	1.487 (2)	C11B—C11B^ii^	1.486 (2)
C12A—C13A	1.3844 (15)	C12B—C13B	1.3834 (15)
C12A—H12A	0.9500	C12B—H12B	0.9500
C13A—H13A	0.9500	C13B—H13B	0.9500
			
C4A—N1A—C5A	114.19 (9)	C5B—N1B—C1B	114.00 (9)
C4A—N1A—C1A	103.63 (8)	C5B—N1B—C4B	114.41 (9)
C5A—N1A—C1A	112.60 (8)	C1B—N1B—C4B	104.43 (9)
N1A—C1A—C2A	102.99 (9)	N1B—C1B—C2B	102.86 (9)
N1A—C1A—H1A1	111.2	N1B—C1B—H1B1	111.2
C2A—C1A—H1A1	111.2	C2B—C1B—H1B1	111.2
N1A—C1A—H1A2	111.2	N1B—C1B—H1B2	111.2
C2A—C1A—H1A2	111.2	C2B—C1B—H1B2	111.2
H1A1—C1A—H1A2	109.1	H1B1—C1B—H1B2	109.1
C1A—C2A—C3A	104.25 (9)	C1B—C2B—C3B	104.18 (9)
C1A—C2A—H2A1	110.9	C1B—C2B—H2B1	110.9
C3A—C2A—H2A1	110.9	C3B—C2B—H2B1	110.9
C1A—C2A—H2A2	110.9	C1B—C2B—H2B2	110.9
C3A—C2A—H2A2	110.9	C3B—C2B—H2B2	110.9
H2A1—C2A—H2A2	108.9	H2B1—C2B—H2B2	108.9
C4A—C3A—C2A	104.34 (9)	C4B—C3B—C2B	104.86 (9)
C4A—C3A—H3A1	110.9	C4B—C3B—H3B1	110.8
C2A—C3A—H3A1	110.9	C2B—C3B—H3B1	110.8
C4A—C3A—H3A2	110.9	C4B—C3B—H3B2	110.8
C2A—C3A—H3A2	110.9	C2B—C3B—H3B2	110.8
H3A1—C3A—H3A2	108.9	H3B1—C3B—H3B2	108.9
N1A—C4A—C3A	103.36 (9)	N1B—C4B—C3B	103.67 (9)
N1A—C4A—H4A1	111.1	N1B—C4B—H4B1	111.0
C3A—C4A—H4A1	111.1	C3B—C4B—H4B1	111.0
N1A—C4A—H4A2	111.1	N1B—C4B—H4B2	111.0
C3A—C4A—H4A2	111.1	C3B—C4B—H4B2	111.0
H4A1—C4A—H4A2	109.1	H4B1—C4B—H4B2	109.0
N1A—C5A—C6A	112.54 (8)	N1B—C5B—C6B	115.14 (9)
N1A—C5A—H5A1	109.1	N1B—C5B—H5B1	108.5
C6A—C5A—H5A1	109.1	C6B—C5B—H5B1	108.5
N1A—C5A—H5A2	109.1	N1B—C5B—H5B2	108.5
C6A—C5A—H5A2	109.1	C6B—C5B—H5B2	108.5
H5A1—C5A—H5A2	107.8	H5B1—C5B—H5B2	107.5
C7A—C6A—C5A	176.79 (11)	C7B—C6B—C5B	177.04 (11)
C6A—C7A—C8A	175.84 (11)	C6B—C7B—C8B	177.29 (11)
C9A—C8A—C13A	118.34 (10)	C9B—C8B—C13B	118.07 (10)
C9A—C8A—C7A	119.38 (10)	C9B—C8B—C7B	120.32 (10)
C13A—C8A—C7A	122.28 (10)	C13B—C8B—C7B	121.61 (10)
C10A—C9A—C8A	120.86 (10)	C10B—C9B—C8B	120.86 (10)
C10A—C9A—H9A	119.6	C10B—C9B—H9B	119.6
C8A—C9A—H9A	119.6	C8B—C9B—H9B	119.6
C9A—C10A—C11A	121.62 (10)	C9B—C10B—C11B	121.74 (10)
C9A—C10A—H10A	119.2	C9B—C10B—H10B	119.1
C11A—C10A—H10A	119.2	C11B—C10B—H10B	119.1
C10A—C11A—C12A	116.84 (10)	C10B—C11B—C12B	116.87 (10)
C10A—C11A—C11A^i^	121.06 (12)	C10B—C11B—C11B^ii^	121.34 (12)
C12A—C11A—C11A^i^	122.10 (12)	C12B—C11B—C11B^ii^	121.79 (11)
C13A—C12A—C11A	121.94 (10)	C13B—C12B—C11B	121.76 (10)
C13A—C12A—H12A	119.0	C13B—C12B—H12B	119.1
C11A—C12A—H12A	119.0	C11B—C12B—H12B	119.1
C12A—C13A—C8A	120.35 (10)	C12B—C13B—C8B	120.68 (10)
C12A—C13A—H13A	119.8	C12B—C13B—H13B	119.7
C8A—C13A—H13A	119.8	C8B—C13B—H13B	119.7
			
C4A—N1A—C1A—C2A	−45.38 (10)	C5B—N1B—C1B—C2B	170.74 (9)
C5A—N1A—C1A—C2A	−169.28 (9)	C4B—N1B—C1B—C2B	45.18 (11)
N1A—C1A—C2A—C3A	27.96 (11)	N1B—C1B—C2B—C3B	−30.78 (12)
C1A—C2A—C3A—C4A	−1.49 (11)	C1B—C2B—C3B—C4B	6.22 (12)
C5A—N1A—C4A—C3A	167.28 (9)	C5B—N1B—C4B—C3B	−166.33 (9)
C1A—N1A—C4A—C3A	44.43 (10)	C1B—N1B—C4B—C3B	−41.02 (11)
C2A—C3A—C4A—N1A	−25.57 (11)	C2B—C3B—C4B—N1B	20.48 (12)
C4A—N1A—C5A—C6A	78.62 (11)	C1B—N1B—C5B—C6B	−62.66 (13)
C1A—N1A—C5A—C6A	−163.55 (9)	C4B—N1B—C5B—C6B	57.45 (13)
C13A—C8A—C9A—C10A	1.59 (15)	C13B—C8B—C9B—C10B	1.28 (15)
C7A—C8A—C9A—C10A	−178.97 (9)	C7B—C8B—C9B—C10B	−177.95 (9)
C8A—C9A—C10A—C11A	0.51 (15)	C8B—C9B—C10B—C11B	−0.73 (16)
C9A—C10A—C11A—C12A	−2.01 (14)	C9B—C10B—C11B—C12B	−0.51 (15)
C9A—C10A—C11A—C11A^i^	178.33 (10)	C9B—C10B—C11B—C11B^ii^	179.42 (11)
C10A—C11A—C12A—C13A	1.47 (15)	C10B—C11B—C12B—C13B	1.19 (15)
C11A^i^—C11A—C12A—C13A	−178.88 (11)	C11B^ii^—C11B—C12B—C13B	−178.74 (11)
C11A—C12A—C13A—C8A	0.58 (16)	C11B—C12B—C13B—C8B	−0.65 (16)
C9A—C8A—C13A—C12A	−2.12 (15)	C9B—C8B—C13B—C12B	−0.60 (15)
C7A—C8A—C13A—C12A	178.46 (9)	C7B—C8B—C13B—C12B	178.61 (9)

Symmetry codes: (i) −*x*, −*y*+1, −*z*+1; (ii) −*x*, −*y*+2, −*z*+1.

### (II) 4,4'-Bis{3-[(S)-2-methylpyrrolidin-1-yl]prop-1-yn-1-yl}-1,1'-biphenyl Crystal data

**Table:**

C_28_H_32_N_2_	*F*(000) = 428
*M**_r_* = 396.55	*D*_x_ = 1.178 Mg m^−^^3^
Monoclinic, *P*2_1_	Mo *K*α radiation, λ = 0.71073 Å
*a* = 8.1411 (4) Å	Cell parameters from 2730 reflections
*b* = 7.3080 (4) Å	θ = 1.0–27.5°
*c* = 18.9840 (9) Å	µ = 0.07 mm^−^^1^
β = 98.177 (3)°	*T* = 90 K
*V* = 1117.97 (10) Å^3^	Cut slab, colourless
*Z* = 2	0.41 × 0.35 × 0.08 mm

### (II) 4,4'-Bis{3-[(S)-2-methylpyrrolidin-1-yl]prop-1-yn-1-yl}-1,1'-biphenyl Data collection

**Table:**

Nonius KappaCCD diffractometer	4705 independent reflections
Radiation source: fine-focus sealed-tube	3548 reflections with *I* > 2σ(*I*)
Detector resolution: 9.1 pixels mm^-1^	*R*_int_ = 0.085
φ and ω scans at fixed χ = 55°	θ_max_ = 27.5°, θ_min_ = 2.2°
Absorption correction: multi-scan (*SADABS*; Krause *et al.*, 2015)	*h* = −10→10
*T*_min_ = 0.791, *T*_max_ = 0.971	*k* = −8→9
15874 measured reflections	*l* = −24→24

### (II) 4,4'-Bis{3-[(S)-2-methylpyrrolidin-1-yl]prop-1-yn-1-yl}-1,1'-biphenyl Refinement

**Table:**

Refinement on *F*^2^	Secondary atom site location: difference Fourier map
Least-squares matrix: full	Hydrogen site location: difference Fourier map
*R*[*F*^2^ > 2σ(*F*^2^)] = 0.054	H-atom parameters constrained
*wR*(*F*^2^) = 0.144	*w* = 1/[σ^2^(*F*_o_^2^) + (0.0742*P*)^2^ + 0.0409*P*] where *P* = (*F*_o_^2^ + 2*F*_c_^2^)/3
*S* = 1.05	(Δ/σ)_max_ < 0.001
4705 reflections	Δρ_max_ = 0.30 e Å^−^^3^
273 parameters	Δρ_min_ = −0.19 e Å^−^^3^
1 restraint	Absolute structure: Flack *x* parameter was determined using 1205 quotients of the form [(*I*^+^)-(*I*^-^)]/[(*I*^+^)+(*I*^-^)] (Parsons *et al.*, 2013)
Primary atom site location: structure-invariant direct methods	Absolute structure parameter: −0.3 (10)

### (II) 4,4'-Bis{3-[(S)-2-methylpyrrolidin-1-yl]prop-1-yn-1-yl}-1,1'-biphenyl Special details

**Table:**

Geometry. All e.s.d.'s (except the e.s.d. in the dihedral angle between two l.s. planes) are estimated using the full covariance matrix. The cell e.s.d.'s are taken into account individually in the estimation of e.s.d.'s in distances, angles and torsion angles; correlations between e.s.d.'s in cell parameters are only used when they are defined by crystal symmetry. An approximate (isotropic) treatment of cell e.s.d.'s is used for estimating e.s.d.'s involving l.s. planes.
Refinement. Refinement progress was checked using *PLATON* (Spek, 2009) and by an *R*-tensor (Parkin, 2000). The final model was further checked with the IUCr utility *checkCIF*.Absolute structure analysis: The Flack x parameter was determined using 1205 quotients of the form [(I+)-(I-)]/[(I+)+(I-)], but since the anomalous signal was so small the result is thoroughly inconclusive. This is to be expected, and merely confirms what we already know about light atom non-centrosymmetric structures that are determined with MoKα radiation. The quotient method has been described by Parsons *et al.* (2013). However, the synthesis used pure (*S*)-2-methylpyrrolidine, so the absolute configuration for the model of (II) was dictated by the synthesis.

### (II) 4,4'-Bis{3-[(S)-2-methylpyrrolidin-1-yl]prop-1-yn-1-yl}-1,1'-biphenyl Fractional atomic coordinates and isotropic or equivalent isotropic displacement parameters (Å^2^)

**Table:**

	*x*	*y*	*z*	*U*_iso_*/*U*_eq_	
N1	−0.0921 (3)	0.5633 (4)	0.18120 (12)	0.0294 (6)	
N2	1.5991 (3)	0.4631 (4)	0.82088 (11)	0.0288 (6)	
C1	−0.0274 (4)	0.7478 (5)	0.17326 (15)	0.0333 (7)	
H1A	0.0846	0.7619	0.2008	0.040*	
H1B	−0.1021	0.8413	0.1892	0.040*	
C2	−0.0213 (4)	0.7632 (5)	0.09382 (16)	0.0384 (8)	
H2A	0.0767	0.8345	0.0845	0.046*	
H2B	−0.1229	0.8226	0.0692	0.046*	
C3	−0.0094 (4)	0.5640 (5)	0.06935 (15)	0.0381 (8)	
H3A	−0.1085	0.5300	0.0354	0.046*	
H3B	0.0907	0.5459	0.0460	0.046*	
C4	0.0011 (3)	0.4496 (5)	0.13712 (15)	0.0327 (7)	
H4A	0.1195	0.4424	0.1599	0.039*	
C5	−0.0680 (5)	0.2582 (6)	0.12600 (18)	0.0533 (10)	
H5A	−0.0562	0.1938	0.1717	0.080*	
H5B	−0.0069	0.1920	0.0931	0.080*	
H5C	−0.1857	0.2647	0.1060	0.080*	
C6	−0.0833 (3)	0.5055 (5)	0.25511 (14)	0.0336 (8)	
H6A	−0.1321	0.3816	0.2560	0.040*	
H6B	−0.1527	0.5892	0.2794	0.040*	
C7	0.0854 (3)	0.5015 (4)	0.29597 (14)	0.0296 (7)	
C8	0.2237 (3)	0.5035 (4)	0.32840 (13)	0.0258 (6)	
C9	0.3832 (3)	0.5132 (4)	0.37271 (13)	0.0251 (6)	
C10	0.5206 (3)	0.4133 (4)	0.35812 (14)	0.0263 (6)	
H10A	0.5128	0.3411	0.3162	0.032*	
C11	0.6689 (3)	0.4188 (4)	0.40464 (13)	0.0249 (6)	
H11A	0.7614	0.3508	0.3937	0.030*	
C12	0.6847 (3)	0.5219 (4)	0.46694 (13)	0.0243 (6)	
C13	0.5480 (3)	0.6270 (4)	0.47988 (14)	0.0254 (6)	
H13A	0.5569	0.7026	0.5210	0.030*	
C14	0.4002 (3)	0.6225 (4)	0.43367 (13)	0.0257 (6)	
H14A	0.3091	0.6947	0.4436	0.031*	
C15	0.8386 (3)	0.5167 (4)	0.51885 (13)	0.0239 (6)	
C16	0.9931 (4)	0.4757 (4)	0.49786 (15)	0.0261 (6)	
H16A	1.0010	0.4593	0.4488	0.031*	
C17	1.1338 (3)	0.4589 (4)	0.54754 (14)	0.0271 (7)	
H17A	1.2369	0.4320	0.5319	0.033*	
C18	1.1278 (3)	0.4805 (4)	0.61967 (13)	0.0247 (6)	
C19	0.9756 (3)	0.5261 (4)	0.64146 (14)	0.0276 (7)	
H19A	0.9690	0.5456	0.6905	0.033*	
C20	0.8343 (3)	0.5429 (4)	0.59141 (14)	0.0271 (7)	
H20A	0.7319	0.5731	0.6070	0.033*	
C21	1.2782 (3)	0.4600 (5)	0.66923 (13)	0.0287 (7)	
C22	1.4103 (3)	0.4380 (5)	0.70559 (14)	0.0318 (7)	
C23	1.5765 (3)	0.4087 (6)	0.74681 (14)	0.0388 (9)	
H23A	1.6034	0.2769	0.7446	0.047*	
H23B	1.6583	0.4760	0.7228	0.047*	
C24	1.5747 (4)	0.6593 (5)	0.83087 (16)	0.0364 (7)	
H24A	1.4666	0.7004	0.8053	0.044*	
H24B	1.6646	0.7315	0.8142	0.044*	
C25	1.5796 (5)	0.6761 (6)	0.91101 (17)	0.0487 (10)	
H25A	1.5039	0.7738	0.9229	0.058*	
H25B	1.6934	0.7034	0.9346	0.058*	
C26	1.5222 (4)	0.4886 (6)	0.93362 (15)	0.0439 (9)	
H26A	1.6096	0.4298	0.9678	0.053*	
H26B	1.4204	0.5003	0.9563	0.053*	
C27	1.4879 (4)	0.3770 (5)	0.86523 (16)	0.0347 (8)	
H27A	1.3706	0.3987	0.8431	0.042*	
C28	1.5151 (4)	0.1744 (5)	0.8746 (2)	0.0535 (10)	
H28A	1.4918	0.1137	0.8282	0.080*	
H28C	1.4408	0.1262	0.9064	0.080*	
H28D	1.6306	0.1513	0.8952	0.080*	

### (II) 4,4'-Bis{3-[(S)-2-methylpyrrolidin-1-yl]prop-1-yn-1-yl}-1,1'-biphenyl Atomic displacement parameters (Å^2^)

**Table:**

	*U*^11^	*U*^22^	*U*^33^	*U*^12^	*U*^13^	*U*^23^
N1	0.0231 (12)	0.0336 (16)	0.0301 (12)	0.0017 (11)	−0.0014 (9)	−0.0003 (11)
N2	0.0211 (12)	0.0362 (16)	0.0283 (12)	−0.0022 (11)	0.0011 (9)	−0.0015 (12)
C1	0.0264 (15)	0.0318 (19)	0.0403 (17)	0.0012 (14)	−0.0002 (12)	0.0003 (15)
C2	0.0305 (17)	0.041 (2)	0.0421 (17)	0.0034 (15)	−0.0011 (13)	0.0105 (16)
C3	0.0361 (17)	0.047 (2)	0.0307 (15)	−0.0027 (16)	0.0015 (12)	−0.0005 (15)
C4	0.0256 (15)	0.038 (2)	0.0336 (15)	0.0000 (14)	−0.0002 (12)	−0.0055 (14)
C5	0.069 (3)	0.039 (2)	0.051 (2)	−0.007 (2)	0.0060 (18)	−0.0085 (18)
C6	0.0225 (14)	0.044 (2)	0.0334 (15)	−0.0034 (14)	0.0028 (11)	0.0003 (15)
C7	0.0272 (15)	0.0339 (19)	0.0274 (13)	0.0008 (13)	0.0034 (11)	0.0004 (14)
C8	0.0279 (14)	0.0249 (17)	0.0246 (13)	0.0006 (12)	0.0036 (11)	0.0004 (12)
C9	0.0249 (14)	0.0244 (17)	0.0259 (13)	−0.0009 (12)	0.0035 (10)	0.0051 (12)
C10	0.0289 (15)	0.0260 (17)	0.0239 (13)	−0.0005 (13)	0.0031 (11)	−0.0023 (12)
C11	0.0223 (14)	0.0268 (17)	0.0262 (13)	0.0014 (12)	0.0053 (10)	0.0038 (12)
C12	0.0206 (13)	0.0245 (17)	0.0274 (13)	−0.0017 (12)	0.0021 (10)	0.0043 (13)
C13	0.0270 (14)	0.0215 (16)	0.0279 (14)	−0.0007 (12)	0.0048 (11)	−0.0027 (13)
C14	0.0224 (13)	0.0252 (17)	0.0299 (14)	0.0030 (12)	0.0044 (11)	0.0011 (13)
C15	0.0196 (13)	0.0209 (17)	0.0304 (13)	−0.0036 (12)	0.0009 (10)	0.0008 (13)
C16	0.0266 (13)	0.0249 (17)	0.0271 (12)	−0.0010 (13)	0.0052 (10)	0.0000 (13)
C17	0.0190 (13)	0.0267 (17)	0.0358 (14)	−0.0007 (12)	0.0047 (11)	0.0023 (13)
C18	0.0221 (13)	0.0191 (16)	0.0318 (14)	−0.0039 (12)	0.0002 (10)	−0.0002 (12)
C19	0.0245 (14)	0.0334 (19)	0.0248 (13)	−0.0003 (13)	0.0024 (10)	−0.0033 (13)
C20	0.0212 (13)	0.0303 (18)	0.0301 (14)	0.0008 (12)	0.0041 (10)	−0.0001 (13)
C21	0.0275 (15)	0.0292 (19)	0.0296 (14)	0.0002 (13)	0.0042 (11)	−0.0007 (13)
C22	0.0273 (15)	0.037 (2)	0.0301 (14)	0.0024 (14)	0.0015 (11)	−0.0019 (14)
C23	0.0223 (15)	0.060 (3)	0.0331 (15)	0.0071 (15)	0.0003 (12)	−0.0025 (16)
C24	0.0350 (16)	0.0319 (19)	0.0395 (17)	−0.0074 (14)	−0.0047 (13)	0.0015 (15)
C25	0.053 (2)	0.048 (3)	0.0425 (19)	−0.0021 (18)	−0.0044 (16)	−0.0102 (17)
C26	0.0386 (18)	0.062 (3)	0.0307 (15)	0.0077 (17)	0.0050 (13)	0.0041 (17)
C27	0.0204 (15)	0.041 (2)	0.0423 (17)	−0.0013 (13)	0.0015 (13)	0.0094 (15)
C28	0.0384 (19)	0.038 (2)	0.079 (3)	−0.0052 (17)	−0.0083 (17)	0.0178 (19)

### (II) 4,4'-Bis{3-[(S)-2-methylpyrrolidin-1-yl]prop-1-yn-1-yl}-1,1'-biphenyl Geometric parameters (Å, º)

**Table:**

N1—C6	1.457 (4)	C13—C14	1.385 (3)
N1—C1	1.463 (4)	C13—H13A	0.9500
N1—C4	1.465 (4)	C14—H14A	0.9500
N2—C23	1.448 (3)	C15—C20	1.396 (4)
N2—C27	1.463 (4)	C15—C16	1.405 (4)
N2—C24	1.464 (4)	C16—C17	1.381 (4)
C1—C2	1.520 (4)	C16—H16A	0.9500
C1—H1A	0.9900	C17—C18	1.386 (4)
C1—H1B	0.9900	C17—H17A	0.9500
C2—C3	1.536 (5)	C18—C19	1.401 (4)
C2—H2A	0.9900	C18—C21	1.442 (3)
C2—H2B	0.9900	C19—C20	1.389 (4)
C3—C4	1.526 (4)	C19—H19A	0.9500
C3—H3A	0.9900	C20—H20A	0.9500
C3—H3B	0.9900	C21—C22	1.203 (4)
C4—C5	1.511 (5)	C22—C23	1.479 (4)
C4—H4A	1.0000	C23—H23A	0.9900
C5—H5A	0.9800	C23—H23B	0.9900
C5—H5B	0.9800	C24—C25	1.521 (4)
C5—H5C	0.9800	C24—H24A	0.9900
C6—C7	1.479 (4)	C24—H24B	0.9900
C6—H6A	0.9900	C25—C26	1.529 (6)
C6—H6B	0.9900	C25—H25A	0.9900
C7—C8	1.204 (3)	C25—H25B	0.9900
C8—C9	1.445 (3)	C26—C27	1.525 (5)
C9—C10	1.396 (4)	C26—H26A	0.9900
C9—C14	1.397 (4)	C26—H26B	0.9900
C10—C11	1.391 (3)	C27—C28	1.504 (5)
C10—H10A	0.9500	C27—H27A	1.0000
C11—C12	1.393 (4)	C28—H28A	0.9800
C11—H11A	0.9500	C28—H28C	0.9800
C12—C13	1.403 (4)	C28—H28D	0.9800
C12—C15	1.480 (3)		
			
C6—N1—C1	113.4 (2)	C13—C14—C9	120.8 (2)
C6—N1—C4	115.3 (2)	C13—C14—H14A	119.6
C1—N1—C4	103.9 (2)	C9—C14—H14A	119.6
C23—N2—C27	115.9 (2)	C20—C15—C16	117.2 (2)
C23—N2—C24	113.2 (3)	C20—C15—C12	121.1 (2)
C27—N2—C24	103.9 (2)	C16—C15—C12	121.6 (2)
N1—C1—C2	103.5 (2)	C17—C16—C15	120.9 (2)
N1—C1—H1A	111.1	C17—C16—H16A	119.5
C2—C1—H1A	111.1	C15—C16—H16A	119.5
N1—C1—H1B	111.1	C16—C17—C18	121.5 (2)
C2—C1—H1B	111.1	C16—C17—H17A	119.3
H1A—C1—H1B	109.0	C18—C17—H17A	119.3
C1—C2—C3	104.0 (3)	C17—C18—C19	118.4 (2)
C1—C2—H2A	111.0	C17—C18—C21	119.1 (2)
C3—C2—H2A	111.0	C19—C18—C21	122.4 (2)
C1—C2—H2B	111.0	C20—C19—C18	120.0 (2)
C3—C2—H2B	111.0	C20—C19—H19A	120.0
H2A—C2—H2B	109.0	C18—C19—H19A	120.0
C4—C3—C2	105.2 (3)	C19—C20—C15	121.9 (2)
C4—C3—H3A	110.7	C19—C20—H20A	119.0
C2—C3—H3A	110.7	C15—C20—H20A	119.0
C4—C3—H3B	110.7	C22—C21—C18	174.2 (3)
C2—C3—H3B	110.7	C21—C22—C23	177.0 (3)
H3A—C3—H3B	108.8	N2—C23—C22	117.0 (2)
N1—C4—C5	113.1 (3)	N2—C23—H23A	108.0
N1—C4—C3	101.5 (3)	C22—C23—H23A	108.0
C5—C4—C3	114.5 (3)	N2—C23—H23B	108.0
N1—C4—H4A	109.2	C22—C23—H23B	108.0
C5—C4—H4A	109.2	H23A—C23—H23B	107.3
C3—C4—H4A	109.2	N2—C24—C25	102.9 (3)
C4—C5—H5A	109.5	N2—C24—H24A	111.2
C4—C5—H5B	109.5	C25—C24—H24A	111.2
H5A—C5—H5B	109.5	N2—C24—H24B	111.2
C4—C5—H5C	109.5	C25—C24—H24B	111.2
H5A—C5—H5C	109.5	H24A—C24—H24B	109.1
H5B—C5—H5C	109.5	C24—C25—C26	104.1 (3)
N1—C6—C7	115.2 (2)	C24—C25—H25A	110.9
N1—C6—H6A	108.5	C26—C25—H25A	110.9
C7—C6—H6A	108.5	C24—C25—H25B	110.9
N1—C6—H6B	108.5	C26—C25—H25B	110.9
C7—C6—H6B	108.5	H25A—C25—H25B	109.0
H6A—C6—H6B	107.5	C27—C26—C25	105.5 (2)
C8—C7—C6	177.9 (3)	C27—C26—H26A	110.7
C7—C8—C9	174.7 (3)	C25—C26—H26A	110.7
C10—C9—C14	118.4 (2)	C27—C26—H26B	110.7
C10—C9—C8	122.5 (2)	C25—C26—H26B	110.7
C14—C9—C8	119.0 (2)	H26A—C26—H26B	108.8
C11—C10—C9	120.4 (2)	N2—C27—C28	113.5 (3)
C11—C10—H10A	119.8	N2—C27—C26	101.9 (3)
C9—C10—H10A	119.8	C28—C27—C26	114.8 (3)
C10—C11—C12	121.5 (3)	N2—C27—H27A	108.8
C10—C11—H11A	119.3	C28—C27—H27A	108.8
C12—C11—H11A	119.3	C26—C27—H27A	108.8
C11—C12—C13	117.7 (2)	C27—C28—H28A	109.5
C11—C12—C15	121.3 (2)	C27—C28—H28C	109.5
C13—C12—C15	121.0 (2)	H28A—C28—H28C	109.5
C14—C13—C12	121.0 (2)	C27—C28—H28D	109.5
C14—C13—H13A	119.5	H28A—C28—H28D	109.5
C12—C13—H13A	119.5	H28C—C28—H28D	109.5
			
C6—N1—C1—C2	170.4 (2)	C11—C12—C15—C16	26.8 (4)
C4—N1—C1—C2	44.5 (3)	C13—C12—C15—C16	−155.0 (3)
N1—C1—C2—C3	−24.3 (3)	C20—C15—C16—C17	1.2 (4)
C1—C2—C3—C4	−3.3 (3)	C12—C15—C16—C17	−175.5 (3)
C6—N1—C4—C5	66.3 (3)	C15—C16—C17—C18	0.4 (4)
C1—N1—C4—C5	−169.0 (3)	C16—C17—C18—C19	−2.1 (4)
C6—N1—C4—C3	−170.6 (2)	C16—C17—C18—C21	179.5 (3)
C1—N1—C4—C3	−45.9 (3)	C17—C18—C19—C20	2.0 (4)
C2—C3—C4—N1	29.5 (3)	C21—C18—C19—C20	−179.6 (3)
C2—C3—C4—C5	151.6 (3)	C18—C19—C20—C15	−0.4 (4)
C1—N1—C6—C7	−59.8 (3)	C16—C15—C20—C19	−1.2 (4)
C4—N1—C6—C7	59.8 (4)	C12—C15—C20—C19	175.5 (3)
C14—C9—C10—C11	2.0 (4)	C27—N2—C23—C22	57.8 (4)
C8—C9—C10—C11	−176.2 (3)	C24—N2—C23—C22	−62.1 (4)
C9—C10—C11—C12	0.5 (4)	C23—N2—C24—C25	172.0 (2)
C10—C11—C12—C13	−2.8 (4)	C27—N2—C24—C25	45.4 (3)
C10—C11—C12—C15	175.5 (2)	N2—C24—C25—C26	−26.8 (3)
C11—C12—C13—C14	2.6 (4)	C24—C25—C26—C27	−0.1 (3)
C15—C12—C13—C14	−175.7 (3)	C23—N2—C27—C28	66.2 (3)
C12—C13—C14—C9	−0.1 (4)	C24—N2—C27—C28	−169.0 (2)
C10—C9—C14—C13	−2.2 (4)	C23—N2—C27—C26	−169.8 (3)
C8—C9—C14—C13	176.1 (3)	C24—N2—C27—C26	−44.9 (3)
C11—C12—C15—C20	−149.7 (3)	C25—C26—C27—N2	26.9 (3)
C13—C12—C15—C20	28.5 (4)	C25—C26—C27—C28	150.0 (3)

## References

[bb1] Bruker (2006). *APEX2* and *SAINT*. Bruker AXS Inc., Madison, Wisconsin, USA.

[bb2] Bruno, I. J., Cole, J. C., Kessler, M., Luo, J., Motherwell, W. D. S., Purkis, L. H., Smith, B. R., Taylor, R., Cooper, R. I., Harris, S. E. & Orpen, A. G. (2004). *J. Chem. Inf. Model.* **44**, 2133–2144. 10.1021/ci049780b 15554684

[bb3] Groom, C. R. & Allen, F. H. (2014). *Angew. Chem. Int. Ed.* **53**, 662–671. 10.1002/anie.201306438 24382699

[bb4] Krause, L., Herbst-Irmer, R., Sheldrick, G. M. & Stalke, D. (2015). *J. Appl. Cryst.* **48**, 3–10. 10.1107/S1600576714022985 PMC4453166 26089746

[bb5] Nonius (1998). *COLLECT*. Nonius BV, Delft, The Netherlands.

[bb6] Otwinowski, Z. & Minor, W. (2006). *International Tables for Crystallography*, Vol. F, ch. 11.4, pp. 226–235. Chester: International Union of Crystallography.

[bb7] Parkin, S. (2000). *Acta Cryst.* A**56**, 157–162. 10.1107/s010876739901497x 10772457

[bb8] Parkin, S. (2013). *CIFFIX*, http://xray.uky.edu/people/parkin/programs/ciffix

[bb9] Parsons, S., Flack, H. D. & Wagner, T. (2013). *Acta Cryst.* B**69**, 249–259. 10.1107/S2052519213010014 PMC3661305 23719469

[bb10] Sheldrick, G. M. (2008). *Acta Cryst.* A**64**, 112–122. 10.1107/S0108767307043930 18156677

[bb11] Sheldrick, G. M. (2015). *Acta Cryst.* C**71**, 3–8.

[bb12] Spek, A. L. (2009). *Acta Cryst.* D**65**, 148–155. 10.1107/S090744490804362X PMC2631630 19171970

[bb13] Wala, E. P., Crooks, P. A., McIntosh, J. M. & Holtman, J. R. (2012). *Anesth. Analg.* **115**, 713–720. 10.1213/ANE.0b013e31825a3c72 PMC4502964 22610850

[bb14] Zheng, G., Zhang, Z., Dowell, C., Wala, E., Dwoskin, L. P., Holton, J. R., McIntosh, J. M. & Crooks, P. A. (2007). *Bioorg. Med. Chem. Lett.*, **21**, 2476–2479. 10.1016/j.bmcl.2011.02.043 PMC3726002 21397497

